# Multiparticulate Drug Delivery Systems Preserve the Efficacy of Reduced-Dose Intermittent Benznidazole Therapy in Experimental Chagas Disease

**DOI:** 10.21203/rs.3.rs-10424380/v1

**Published:** 2026-08-28

**Authors:** Sebastian Del Rosso, Gastón Bergero, Yanina Luciana Mazzocco, Luciana Mezzano, Mónica C. García, Maria Pilar Aoki

**Affiliations:** Consejo Nacional de Investigaciones Científicas y Técnicas (CONICET), Centro de Investigación en Bioquímica Clínica e Inmunología (CIBICI); Consejo Nacional de Investigaciones Científicas y Técnicas (CONICET), Centro de Investigación en Bioquímica Clínica e Inmunología (CIBICI); Consejo Nacional de Investigaciones Científicas y Técnicas (CONICET), Centro de Investigación en Bioquímica Clínica e Inmunología (CIBICI); Universidad Nacional de Córdoba, Instituto de Biología Celular; Consejo Nacional de Investigaciones Científicas y Técnicas (CONICET), Unidad de Investigación y Desarrollo en Tecnología Farmacéutica (UNITEFA); Consejo Nacional de Investigaciones Científicas y Técnicas (CONICET), Centro de Investigación en Bioquímica Clínica e Inmunología (CIBICI)

## Abstract

Chagas disease, caused by the *Trypanosoma cruzi* parasite infection, is primarily treated with benznidazole (BZ), though continuous dosing regimens are limited by adverse effects that compromise adherence. We evaluated whether a reduced-dose intermittent BZ regimen (50 mg/kg every 2 days, 20 doses), administered as free drug or incorporated into a BZ-loaded multiparticulate drug delivery system (BZ-MDDS), with or without adjunctive CD73 inhibition (APCP), maintains therapeutic efficacy and improves tissue-level safety in an experimental model of infection. Survival, parasitemia, cardiac parasite load, biochemical injury markers, and hepatic histology were assessed at 120 days post-infection (dpi), while echocardiographic parameters were recorded at 105 dpi. The reduced regimen markedly improved survival relative to untreated controls (*p* = 0.0001), with comparable terminal parasitological control across treated groups regardless of formulation or APCP co-administration. BZ-MDDS was associated with more preserved hepatic morphology and lower glutamate pyruvate transaminase elevation than free BZ (*p* < 0.05). Most cardiac, hepatic, and organ-weight parameters showed no significant differences between treated groups; pulmonary/aortic Doppler flow indices and visceral adipose tissue mass were the only parameters that differed, being lower in some treated groups relative to non-infected controls (*p* < 0.05 to *p* < 0.001). Together, these findings indicate that BZ-MDDS maintains the efficacy of a reduced-dose intermittent regimen with a favorable hepatic safety profile, although direct comparison with continuous standard-dose BZ was outside the scope of this study.

## 2. INTRODUCTION

Chagas disease (CD), also known as *American trypanosomiasis*, is a chronic systemic parasitic infection caused by the protozoan *Trypanosoma cruzi* (*T. cruzi*). Epidemiological data indicate that CD is endemic across extensive regions from the southern USA to Latin America [1], with approximately 75 million individuals living at risk of infection [2]. Additionally, migration patterns have led to a significant increase in cases in formerly nonaffected areas, including Europe, Japan, Australia and Canada [3]. Moreover, estimates from different reports suggest a global prevalence of 5.7 to 6.3 million infected individuals with an annual incidence ranging from ~40,000 to over 150,000 new infections per year [2, 4].

The clinical course of CD is generally divided into acute, indeterminate, and chronic phases [5, 6] with the majority (60%–70%) of infected individuals remaining asymptomatic throughout their lifetime despite persistent seropositivity. However, approximately 30% of infected individuals progress from the indeterminate to the chronic (clinical) phase characterized by subpatent parasitemia, tissue parasitism, and a persistent low-intensity inflammatory response. Current estimates indicate a substantially higher mortality rate in CD patients [7] with the annual mortality risk in chronic Chagas cardiomyopathy (CCD) primarily attributable to cardiovascular causes [8]. In this context, the search for effective treatments has remained a central focus of CD research for decades. Despite ongoing efforts, the need for novel and alternative therapeutic strategies persists.

Two drugs are available for the trypanocide treatment of CD. Nifurtimox (NF; 3-methyl-4-[59nitrofurfurylideneamine] tetrahydro-4H-1,4-tiazine-1,1-dioxide) and benznidazole (BZ; N-benzyl-2-nitroimidazole acetamide). They are reasonably effective in both acute and chronic disease [9], although treating chronically infected patients with cardiomyopathy does not affect progression to heart failure [6, 10]. Among the two available drugs, NF has been progressively discontinued because of its high toxicity, leaving BZ as the primary treatment option in most Latin American countries. The need for high doses of BZ—due to its limited gastrointestinal absorption—along with prolonged treatment duration (typically 60 days) and frequent adverse effects, highlights the urgent need for alternative therapeutic strategies. In this sense, several authors have suggested that reducing the BZ dose could be beneficial, as it is associated with a substantial decrease in several side effects [11–14]. The development of innovative strategies employing new drug-controlled-release systems is an attractive approach to improving CD therapy. Our group has reported that multiparticulate drug delivery systems (MDDSs) constituted by interpolyelectrolyte complexes based on Eudragit EPO-Eudragit L100 loaded with BZ, sustain the release of the drug into the circulation and exhibit significant improvements in experimental CD treatment [12]. Compared with the standard free BZ treatment (100 mg/kg/day), BZ-loaded MDDS improved the pharmacotherapy of experimental CD. This approach enhances efficacy even at lower doses and reduces the dosing frequency, improving safety while maintaining effectiveness against *T. cruzi* infection [12].

In addition to standard drug-based therapies, targeting key regulators of the immune response could provide an adjunctive strategy to develop novel treatment alternatives for CD. In this context, purinergic signaling appears to play a central role in various parasitic infections [15]. Following infection, the hypoxic and inflammatory environments induce the release of ATP (eATP) into the extracellular compartment, where eATP triggers important microbicidal effects. However, subsequent action of the CD39 and CD73 ectoenzymes hydrolyzes eATP to adenosine, a potent immunosuppressor. We have shown that CD73 inhibition very early after infection by adenosine 5’-α, β-(methylene) diphosphate (APCP) improved the outcome of CCD by reducing myocardial-specific injury marker (CK-MB) levels and improving electrocardiographic parameters [16], with a concomitant bias toward the M1 macrophage profile in APCP-treated mice. Thus, combining CD73 inhibition with reduced-dose BZ-based regimens provides a biologically plausible strategy to explore whether purinergic modulation influences treatment outcome in experimental *T. cruzi* infection. Accordingly, we investigated whether a reduced-dose intermittent BZ regimen administered as free drug or as a BZ-loaded MDDS, with or without adjunctive CD73 inhibition by APCP, differentially affected survival, parasite persistence, and late tissue- and cardiac-related outcomes in experimental *T. cruzi* infection.

## 3. METHODS

### 3.1. Materials: Drug, polymers and reagents

The BZ was extracted and purified from commercially available tablets (Radanil^®^, Roche, Argentina), as previously reported [12]. Two polymethylacrylates kindly supplied by Etil-pharma (Buenos Aires, Argentina), Eudragit^®^ EPO and Eudragit^®^ L100, were used to develop the MDDS. Physiological solutions (sodium chloride 0.9% w/v) were prepared according to USP specifications (U.S. Pharmacopoeial Convention, 2015), using analytical grade reagents. Cyclophosphamide (Kemex^®^) freeze-dried was reconstituted in physiological solution. Isoflurane (Forane^®^, Abbott, fractioned in Argentina), buffer phosphate solution (PBS, Gibco^®^, Invitrogen), and TRIzol (Thermo Fisher Scientific^®^) were used as purchased.

### 3.2. Preparation of BZ-loaded Multiparticulate Drug Delivery Systems

The methodology used to prepare BZ-loaded MDDSs can be found elsewhere [12, 17]. Briefly, BZ-loaded MDDSs were prepared via a two-step process: a casting solvent, followed by wet granulation. Briefly, the solids of the two PEs (Eudragit EPO (EE) and Eudragit L100 (EL)) and BZ were put in contact in a mortar. EL (0.65 g) was added in stoichiometric proportions to neutralize all ionizable groups of EE (3.15 mmol of equivalents of amino groups per gram of polymer). BZ (1.65 g) was added at a ratio of 1:1 w/w with respect to the total amount of the interpolyelectrolyte complex. Water (w) and hydroalcoholic solvent (h) (1:1 H_2_O:CH_3_CH_2_OH) were used as interaction media. After 24 h, the interpolyelectrolyte complexes loaded with BZ were dried until a constant weight was achieved. Two different BZ-loaded interpolyelectrolyte complexes were subsequently obtained, namely EE-EL-BZw and EE-EL-BZh. A combined BZ-MDDS was prepared using 25% of the total BZ dose from EE-EL-BZw MDDS and the remaining 75% from EE-EL-BZh MDDS.

### 3.3. Animals and ethics statement

Eight-week-old male BALB/c mice were purchased from the Instituto de Ciencias Veterinarias del Litoral (ICIVET-CONICET) (Facultad de Ciencias Veterinarias, Universidad Nacional del Litoral, Argentina). The mice were housed in the Animal Facility of the CIBICI-CONICET (OLAW-NIH assurance number F16–00193). The animals were not subjected to any drug treatment prior to the experiments, and they had *ad libitum* access to water and feed in all the experiments. All animal experiments and procedures were performed with approval of the Committee for Animal Care and Use of the Facultad de Ciencias Químicas, Universidad Nacional de Córdoba, Argentina (Ethical Committee Approval Number Res. HCD 753/14) in strict accordance with the recommendations of the Guide to the Care and Use of Experimental Animals published by the Canadian Council on Animal Care.

### 3.4. Experimental infection and treatments

**Figure 1:**
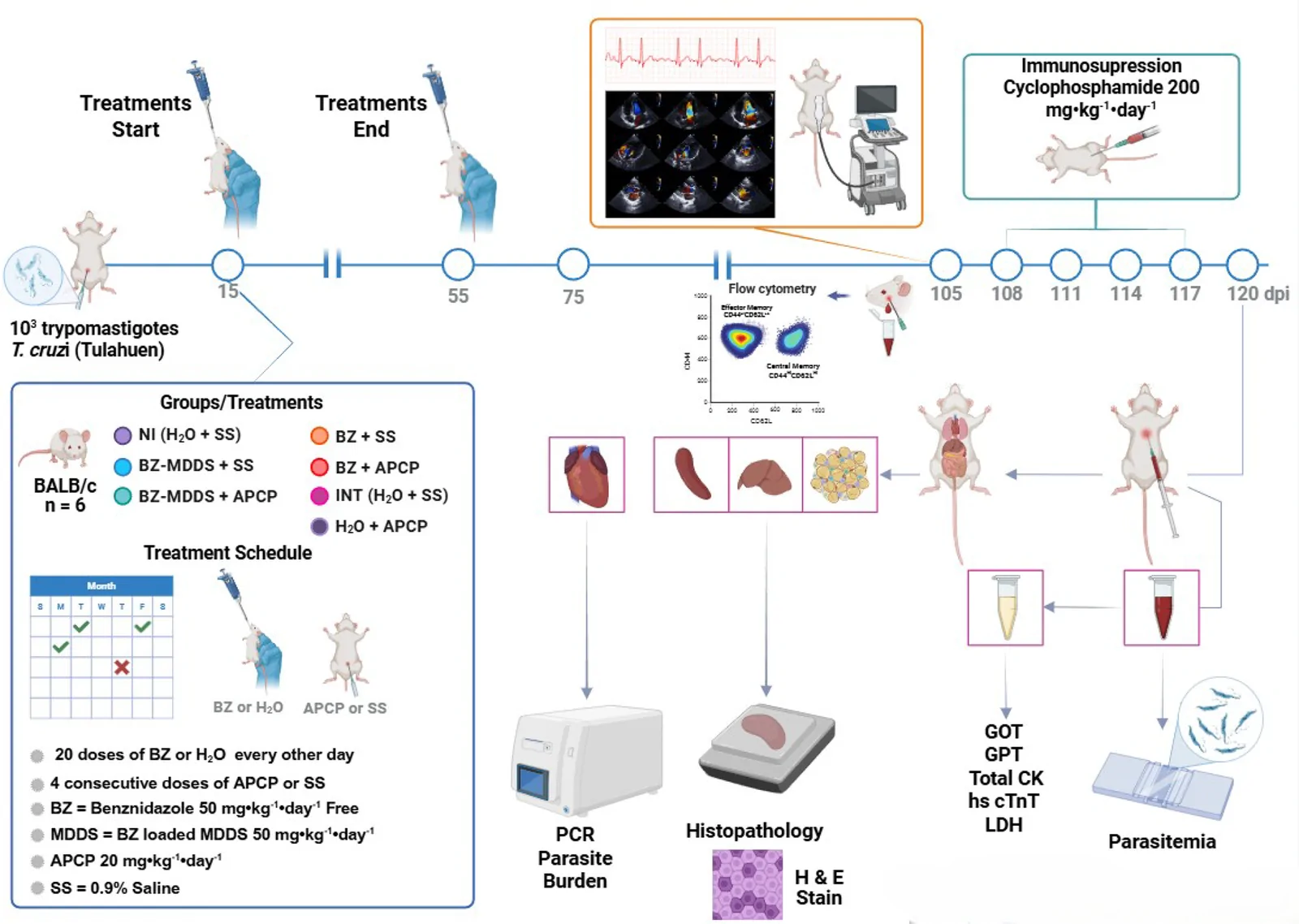
Experimental Protocol. INT = infected not treated, NI = not infected, BZ = Benznidazole, MDDS = Multiparticulate drug delivery system, GOT = glutamic oxaloacetic transaminase, GPT = glutamic pyruvic transaminase, CK = creatine kinase, LDH = lactate dehydrogenase, hs-cTnT = high-sensitivity cardiac troponin T.

BALB/c mice were randomly assigned to the experimental groups (n = 6 per group). Mice assigned to infected groups were infected intraperitoneally (i.p.) with 1,000 blood-derived trypomastigotes of *T. cruzi* (Tulahuen strain). Bloodstream trypomastigote forms were harvested from infected mice via heart puncture and maintained for successive passages. Each mouse was infected with an inoculum from the same stock of parasite-containing solution. Infected mice were monitored daily for the presence of back bristling hair, a continuous tremor in the whole body, and partial loss of mobility of the rear extremities, which usually occurred near 14–15 dpi. Noninfected animals (NI) and infected untreated mice (INT) were used as controls. For all therapy schedules, solid treatment doses were dispersed in 30 μL of water and administered by oral gavage, with the first dose starting at 15 dpi. The experimental groups were: NI; INT; BZ + SS and BZ-MDDS + SS, which received free BZ or BZ-MDDS, respectively, at 50 mg/kg/day every 2 days (twenty alternate-day doses were administered) plus intraperitoneal saline for 4 consecutive days; BZ+APCP and BZ-MDDS+APCP, which received the same oral BZ or BZ-MDDS regimen plus intraperitoneal APCP at 20 mg/kg/day for 4 consecutive days; and H_2_O+APCP, which received water by oral gavage plus the same APCP regimen. NI and INT mice received water by oral gavage and saline solution i.p. After treatment, the animals were kept under daily supervision until 120 dpi, when they were euthanized by deep isoflurane anesthesia. One animal from the NI group was lost for reasons unrelated to the experimental study; therefore, terminal analyses in this group were performed with a final n = 5. A summary of the entire protocol is shown in Fig. 1.

### 3.5. Measurement of treatment efficacy

For the assessment of treatment efficacy, the mice were immunosuppressed by i.p. administration of 200 mg/kg/day cyclophosphamide at 3 day intervals for a total of 4 doses [11, 18], starting at 105 dpi. After immunosuppression, parasitemia was determined by using a Neubauer chamber, with blood samples taken from intracardiac puncture, after incubation with ammonium-chloride-potassium (ACK, Gibco, Lot. 2445679) lysis buffer for 10 min. For determination of tissue parasitism, genomic DNA was purified from cardiac tissues via TRIzol reagent and following the manufacturer’s instructions. Satellite DNA from T. cruzi (GenBank AY520036) was quantified via real time PCR using specific Custom Taqman Gene Expression Assay (Applied Biosystem^®^) and the primers and probe sequences described by Piron et al. [19] (Macrogen Humanizing Genomics, Forward: TGAATGGYGGGAGTCAGAG, Reverse: ATTCCTCCAAGMAGCGGAT, Probe: CACACACTGGACACCAA). A sample containing 2 μg of genomic DNA was amplified. The abundance of satellite DNA from *T. cruzi* was normalized to the GAPDH housekeeping gene control abundance (Mouse GAPD (GAPDH) Endogenous Control probe, Applied Biosystem^®^). For each sample, ΔCt was calculated as Ct(*T. cruzi*) - Ct(GAPDH). The median ΔCt of the BZ+APCP group was used as the internal calibrator. ΔΔCt was calculated as the ΔCt of each sample minus the median ΔCt of the BZ+APCP group. Results were expressed as - ΔΔCt, corresponding to log2 relative quantity in arbitrary units.

### 3.6. Record the Weight of Relevant Organs

After sacrifice, the mice were weighed and perfused with cold PBS for 5 min. The liver, heart, spleen and visceral adipose tissue (VAT) were weighed and normalized to the total body weight.

### 3.7. Tissue Injury Markers

Blood samples were centrifuged at 2500 rpm for 5 min, and the obtained serum was subjected to biochemical determination. Serum samples were analyzed to determine glutamate oxaloacetate transaminase (GOT) and glutamate pyruvate transaminase (GPT) levels and the De Ritis index (calculated as the GOT/GPT ratio) (liver injury markers) [13, 20], creatine kinase (CK), lactate dehydrogenase (LDH) and high-sensitivity cardiac troponin T (hs-cTnT) [21, 22]. These biochemical assays were outsourced to Centro Integral de Bioquímica Especializado en Veterinaria (CIBEV, Cordoba, Argentina).

### 3.8. Liver Histology and Inflammatory Indexes

Livers were fixed in 10% buffered formalin and embedded in paraffin. Five-micron-thick sections were stained with hematoxylin-eosin. Photographs were taken using a Nikon Eclipse TE2000-U using a 20X objective. Inflammation was evaluated semi-quantitatively on low-power field (200X magnification) microscopic examination according to the distribution and extent of inflammatory foci (focal, confluent, or diffuse). Each tissue slide was analyzed for 10 random histological fields, and three different sections per mouse were examined. The presence of inflammatory cells, as well as morphological analysis of liver tissue damage was scored as – (absent), 1+ (focal), 2+ (mild), 3+(extensive inflammatory foci, minimal necrosis, and retention of tissue integrity), or 4+ (diffused inflammation with severe tissue necrosis, interstitial edema, and loss of integrity). Inflammatory infiltrates were characterized as diffused or focal depending upon how closely the inflammatory cells were located [20, 23]. Histological assessment was performed blinded to treatment allocation

### 3.9. Echocardiography and Electrocardiography

At 105 dpi, before immunosuppression, echocardiographic (ECC) and electrocardiographic (ECG) measures were obtained with the animals in a sedated state using a single dose (i.p) of a xylazine (8 mg/kg) and ketamine (90 mg/kg) mixture, adjusted to maintain a heart rate between 200 and 400 bpm. Surface ECG was recorded using a non-invasive TM-300-V device (Temis Tech, Argentina) with a four-electrode configuration that digitally reconstructed the six limb leads (I, II, III, aVR, aVL, and aVF) through the EasyG acquisition software (Temis Tech, Argentina). Recordings were acquired at a paper speed of 50 mm/s and a sensitivity of 40 mm/mV, with filter settings of 1 Hz for baseline correction, 75 Hz for bandwidth limitation, and 40 Hz for muscle noise reduction. ECG analysis was performed offline and was qualitative rather than interval-based. Tracings were examined for abnormalities classically associated with experimental CD, including sustained rhythm disturbances, recurrent ectopic activity, gross atrioventricular or intraventricular conduction defects, abnormal QRS morphology, and consistent ST-T/repolarization abnormalities. Isolated doubtful or nonrecurrent events were interpreted cautiously and were not considered group-defining findings. All echocardiograms were obtained using a LOGIQ e PRO R8 Color Doppler Echocardiography system (General Electric) equipped with a 6.7–18.0 MHz linear array transducer (L8–18i-RS; General Electric). After application of a nontoxic gel, B-mode and M-mode images were recorded. Fractional shortening (FS) was measured in both 2D and M-mode from parasternal long-axis and short-axis views by determining left ventricular end-diastolic and end-systolic diameters; values were averaged from at least three consecutive cardiac cycles. Pulsed Doppler recordings were used to obtain aortic and pulmonary velocity-time integrals (VTIs), as well as mitral inflow E- and A-wave velocities. In the apical five-chamber view, transmitral inflow and transaortic outflow were used to determine isovolumetric contraction time, isovolumetric relaxation time, and ejection time. These measurements were used to calculate the left ventricular myocardial performance index (LVMPI), also known as the TEI index. Doppler-derived parameters were calculated only from recordings with adequate spectral definition and flow alignment, and the TEI index was computed only when all required time intervals could be clearly identified.

### 3.10. Statistical analysis

Descriptive statistics are presented as means ± standard deviations (SD) or medians with interquartile ranges (IQRs), depending on the distribution of the data. The normality of continuous variables was assessed via the Shapiro–Wilk test, complemented by visual inspection of Q–Q plots. Pairwise comparisons were conducted using independent samples t–tests for normally distributed variables, or Mann–Whitney U tests for those with nonnormal distributions. For multiple group comparisons, one-way ANOVA with Fisher’s post-hoc tests were performed or the Kruskal–Wallis tests with Dunn’s post-hoc comparisons were used in the case of nonnormal variables. Survival from infection to 120 dpi was analyzed by the Kaplan–Meier method, and overall differences among groups were assessed using the log-rank (Mantel–Cox) test. When appropriate, post hoc pairwise comparisons were performed between groups. Given that multiple biochemical and echocardiographic endpoints were evaluated independently within each panel, p-values from between-group comparisons within each domain were additionally corrected for multiple testing using the Benjamini-Hochberg false discovery rate procedure. All the statistical analyses were performed using R software [version 4.3.2; R Core Team [24]], and the figures were created with GraphPad Prism (version 9.0 for Windows, GraphPad Software, Boston, MA, USA). All statistical tests were two-sided and statistical significance was predefined at an alpha level of 0.05 for all analyses.

## 4. RESULTS

### Free BZ and BZ-MDDS were associated with high survival under the reduced-dose intermittent regimen.

**Figure 2:**
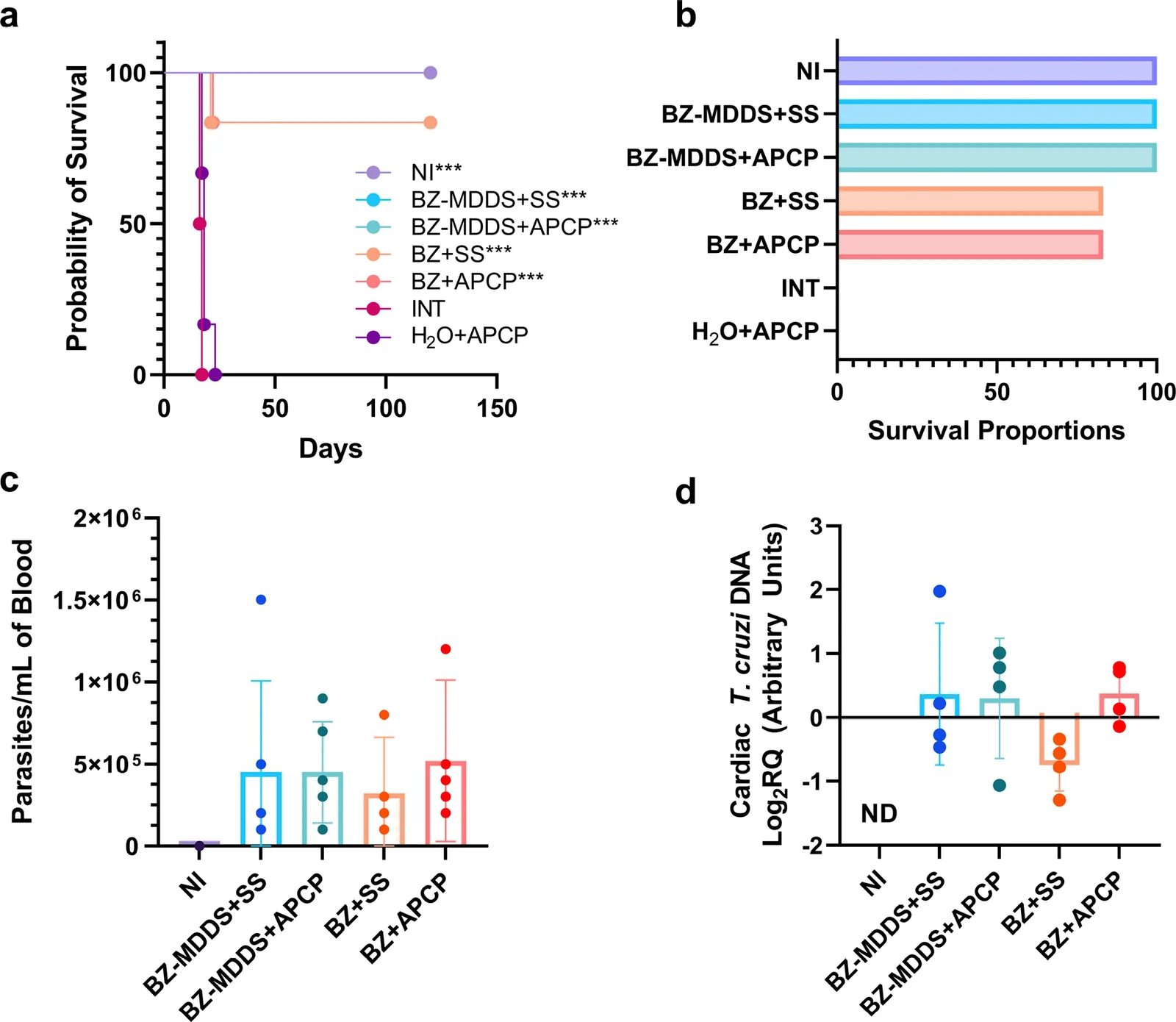
Survival analysis and parasitemia. (a) Kaplan-Meier survival curves displaying the probability of survival for each group. (b) Survival proportions obtained in (a). (c) Blood parasite levels at 120 dpi. (d) Heart parasite load at 120 dpi (*T. cruzi* DNA was not detected in non-infected mice). Survival rate analysis was performed using the Log-rank (Mantel-Cox) test, χ2 = 30.88, df = 6, p-value = 0.0001. Pairwise comparisons, *** p < 0.001 vs INT and H_2_O+APCP. Parasitemia is expressed as medians with interquartile ranges. Statistical comparisons between groups were performed using the Kruskal–Wallis test followed by Dunn’s post hoc analysis. Survival analysis included n = 6 mice per group at study initiation. For terminal parasitemia and cardiac parasite load, n = 5 for NI, BZ+SS, and BZ+APCP, and n = 6 for BZ-MDDS+SS and BZ-MDDS+APCP.

To determine whether therapeutic benefit was retained under a lower BZ burden, infected mice were treated with BZ at 50 mg/kg every 2 days for a total of 20 doses, either as free drug or loaded into the MDDS, in the presence or absence of APCP. As shown in Fig. 2a-b, all treated infected groups survived significantly better than INT mice and those treated only with APCP (log-rank Mantel-Cox: χ^2^ = 30.88, df = 6, *p* = 0.0001; all pairwise comparisons vs INT, *** *p* < 0.001), whereas no differences were detected among treatments or between treated groups and NI animals. Thus, even under a reduced-dose intermittent regimen, both free BZ and BZ-MDDS were sufficient to preserve a marked survival benefit. Because INT mice and H_2_O+APCP-treated mice did not survive to 120 dpi, terminal parasitemia and cardiac parasite load could only be compared with NI and among surviving treated groups; therefore, these late endpoints do not allow direct discrimination between infection-related and treatment-related effects. Under these conditions, we also examined whether APCP co-administration affected these terminal parasitological endpoints. As shown in Fig. 2c-d, there was not a clear APCP-associated separation within either the free BZ or the BZ-MDDS regimens at 120 dpi. Specifically, terminal blood parasitemia and cardiac *T. cruzi* DNA remained broadly comparable between APCP-treated and non-APCP-treated mice within each BZ formulation.

### Selective hepatic biochemical alterations remain detectable despite broadly preserved cardiac and general tissue injury markers.

**Figure 3:**
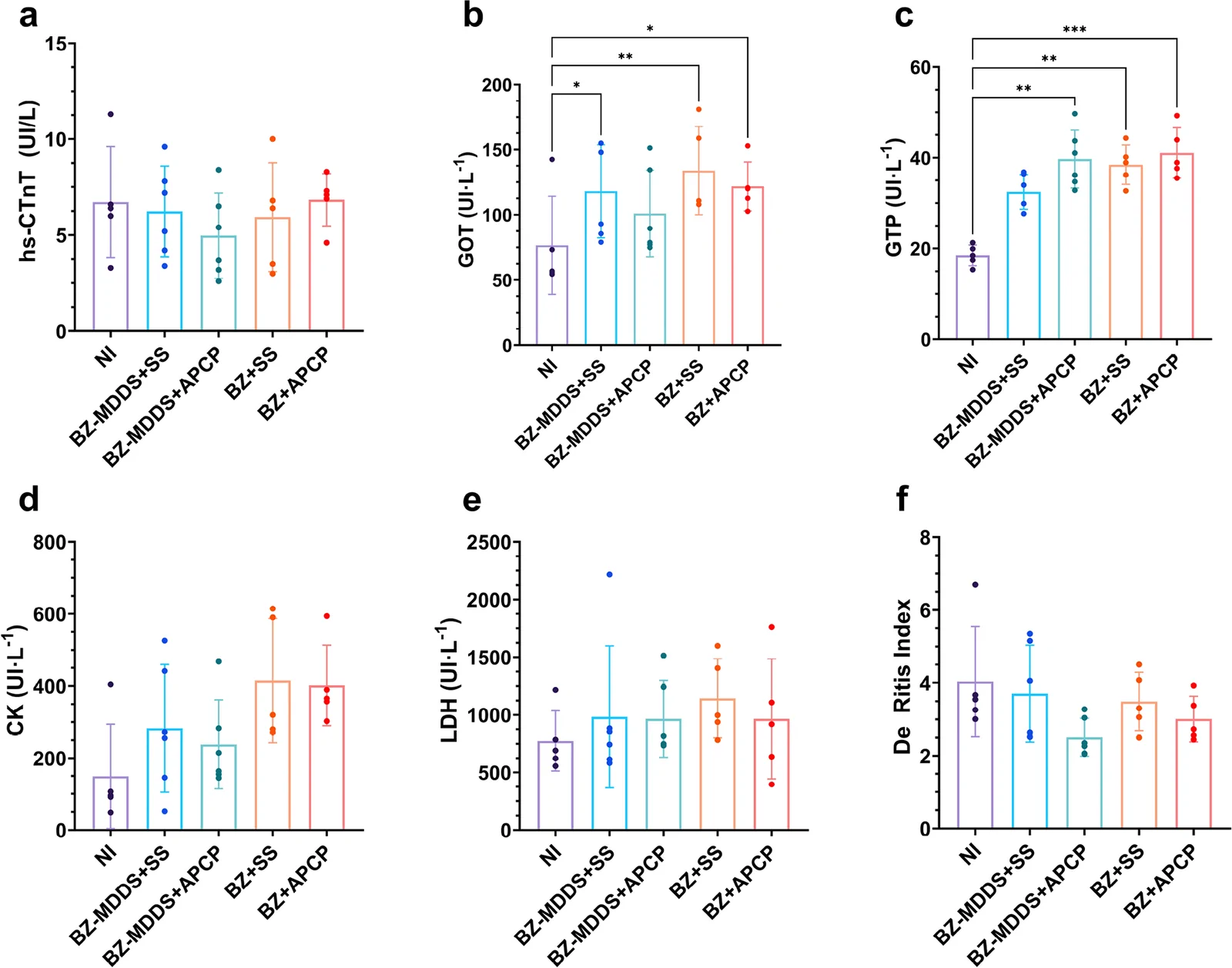
Serum concentrations of tissue damage markers. (a) high-sensitivity cardiac troponin T (hs-cTnT), (b) glutamate oxaloacetate transaminase (GOT), (c) glutamate pyruvate transaminase (GPT), (d) creatine kinase, (e) lactate dehydrogenase, and (f) De Ritis Index. NI = Non-infected, MDDS = Multiparticulate drug delivery system, BZ = benznidazole, APCP = Adenosine 5′-(α,β-methylene)diphosphate. Data are shown as median and interquartile range. The groups were compared using the Kruskal-Wallis test, followed by Dunn’s post-hoc comparisons and corrected for multiple testing using the Benjamini-Hochberg false discovery rate procedure. * P < 0.05. n = 5 for NI, BZ+SS, and BZ+APCP, and n = 6 for BZ-MDDS+SS and BZ-MDDS+APCP.

As shown in Fig. 3a-f, high-sensitivity cardiac troponin T (hs-cTnT), GOT, De Ritis Index, CK, and LDH did not differ significantly among groups, indicating that under this reduced-dose intermittent regimen no major between-group differences were evident in overt cardiac injury or generalized tissue damage. Strikingly, GPT levels (Fig. 3c) were significantly higher in the BZ-MDDS+APCP, BZ + SS, and BZ+APCP groups than in NI mice (*p* < 0.05, significance retained after false discovery rate correction for multiple endpoints), whereas BZ-MDDS + SS remained statistically comparable to the non-infected condition. Thus, within the limits imposed by the absence of a surviving INT group at 120 dpi, GPT emerged as the most sensitive biochemical marker of residual tissue involvement in the present setting. This pattern is directionally consistent with our previous study Garcia, et al. [12], in which hepatic injury markers behaved as informative safety readouts and BZ-MDDS tended to show a more favorable hepatic profile than free BZ.

### Relative heart, spleen, and liver weights remained preserved, whereas visceral adipose tissue was selectively reduced in treated mice.

**Figure 4:**
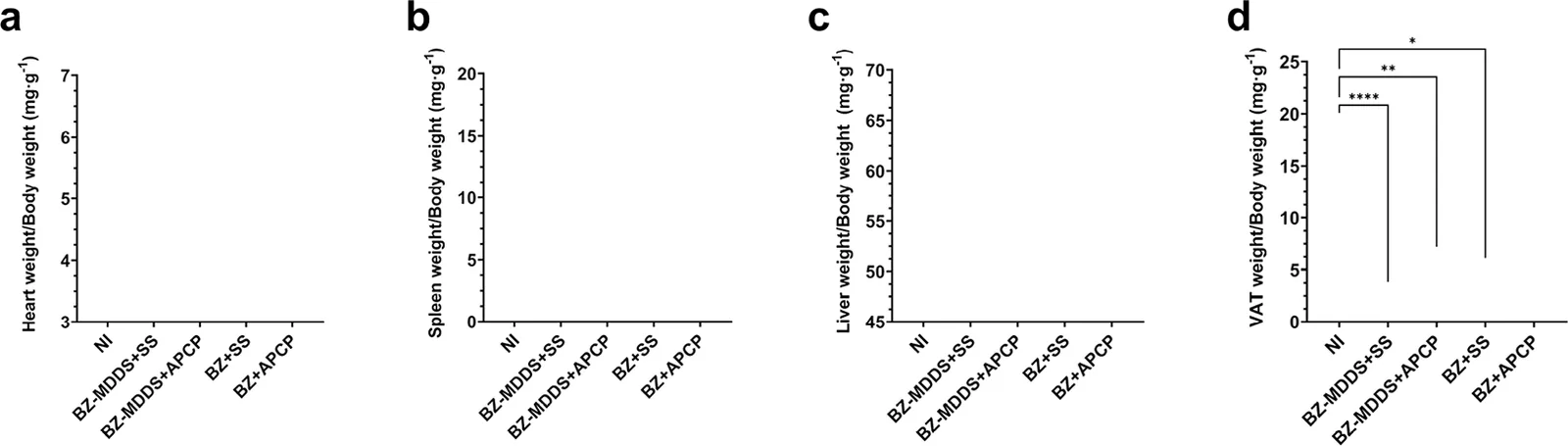
Relative organ weights by the experimental treatments: (a) heart (b) spleen (c) liver (d) visceral adipose tissue. NI = Non-infected, MDDS = Multiparticulate drug delivery system, BZ = benznidazole, APCP = Adenosine 5′-(α,β-methylene)diphosphate. Data are expressed as medians with interquartile ranges. Statistical comparisons between groups were performed using the Kruskal–Wallis test followed by Dunn’s post hoc analysis. *P < 0.05, **P < 0.01, ***P<0.001. n = 5 for NI, BZ+SS, and BZ+APCP, and n = 6 for BZ-MDDS+SS and BZ-MDDS+APCP.

As shown in Fig. 4a-c, no significant differences were detected among groups in relative heart, spleen, or liver weights, indicating that under the present reduced intermittent regimen neither free BZ nor BZ-MDDS induced major late changes in these gross organ-weight parameters. In contrast, Fig. 4d shows that visceral adipose tissue was the only compartment showing significant between-group variation, with significantly lower values in BZ-MDDS + SS, BZ-MDDS+APCP, and BZ + SS relative to NI (*p* < 0.05 to *p* < 0.001). This finding may be biologically plausible, since *T. cruzi* exhibits adipose tissue tropism and adipose tissue has been proposed as a parasite reservoir in both experimental models and humans, while infection has also been associated with reduced fat mass and altered adipocyte biology [25, 26]; however, because adipose parasitism was not directly assessed in the present study, this interpretation should be considered tentative.

### Conventional echocardiographic indices remained preserved, although selected flow-derived parameters were reduced in some treatment groups.

**Figure 5:**
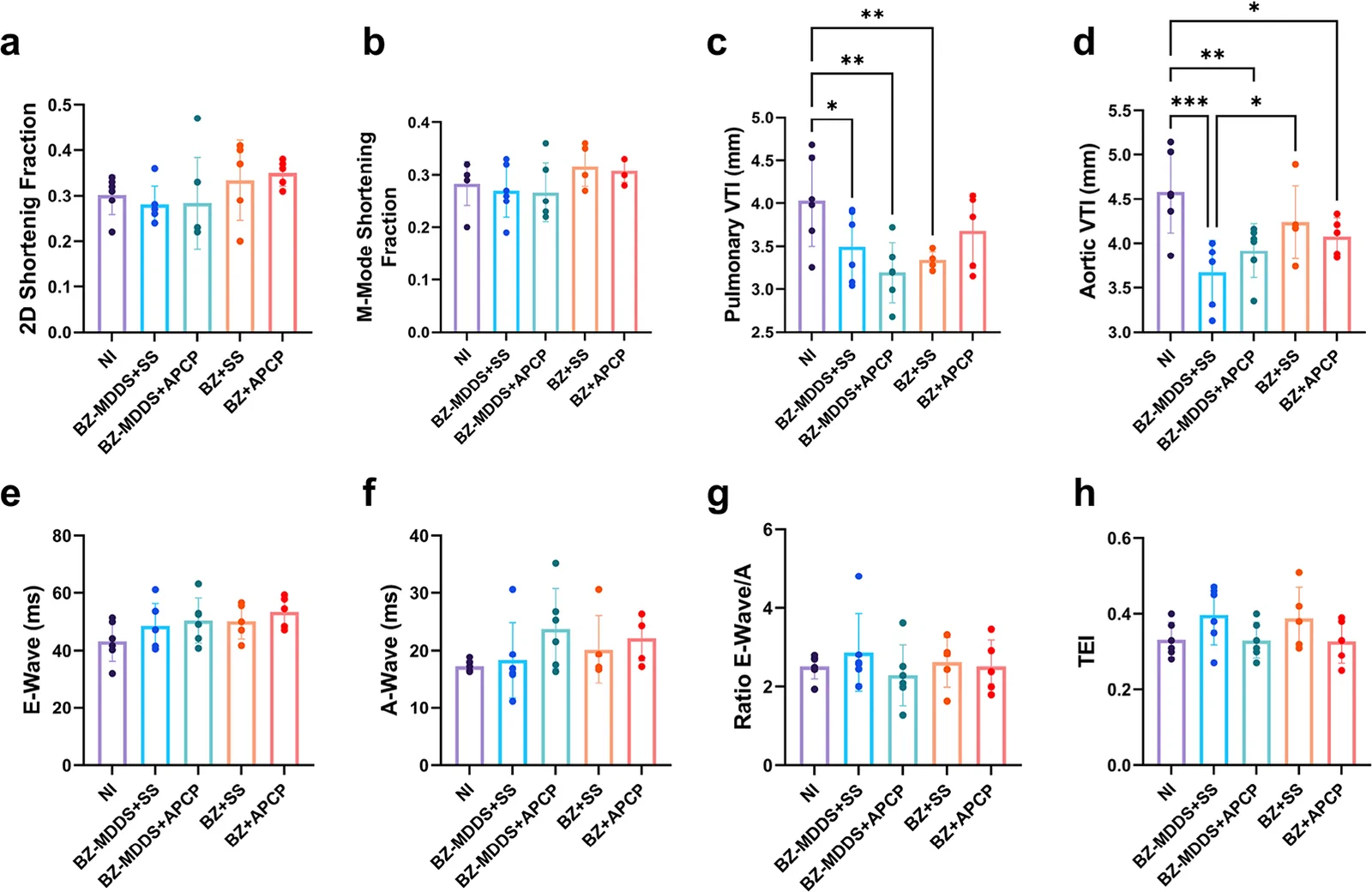
Cardiac function was assessed by echocardiography. (a, b) Fractional shortening was measured using 2D and M-mode imaging, respectively. (c, d) Pulmonary and aortic velocity time integrals (VTIs) were recorded. (e-f) Mitral inflow was evaluated by measuring E and A wave velocities. (g) E/A ratio. (h) The myocardial performance index (Tei index) was calculated as (isovolumetric contraction time + isovolumetric relaxation time) / ejection time. NI = Non-infected, MDDS = Multiparticulate drug delivery system, BZ = benznidazole, APCP = Adenosine 5′-(α,β-methylene)diphosphate. Data are expressed as means and standard deviations. Statistical comparisons between groups were performed using a one-way ANOVA followed by Fisher’s post hoc analysis and corrected for multiple testing using the Benjamini-Hochberg false discovery rate procedure. *p < 0.05, **p < 0.01. n = 6 for NI, BZ-MDDS+SS, and BZ-MDDS+APCP, and n = 5 for BZ+SS and BZ+APCP.

As shown in Fig. 5a, b, e-h, no significant differences were detected among groups in 2D fractional shortening, M-mode fractional shortening, mitral E-wave and A-wave velocities, the E/A ratio, or the Tei index, indicating that under the present reduced-dose intermittent BZ regimen conventional indices of systolic and global myocardial function were largely preserved. By contrast, Fig. 5c-d showed significantly lower pulmonary and aortic velocity-time integrals in BZ-MDDS + SS, BZ-MDDS+APCP, and BZ+APCP relative to NI. Thus, the main functional signal detected in the present setting was not overt impairment of conventional echocardiographic parameters, but rather a reduction in selected Doppler-derived flow indexes. This interpretation is compatible with the broader literature indicating that echocardiographic and Doppler techniques can detect subtle functional abnormalities in CD even when conventional systolic measurements remain within preserved ranges [27, 28]. Because echocardiographic parameters were not evaluated in our previous BZ-MDDS study [12] or in our earlier APCP study [16], the present findings add a functional dimension to the current model, although the biological significance of these lower VTI values should be interpreted cautiously.

In addition, qualitative six-lead ECG assessment did not reveal a consistent pattern of Chagas-compatible electrocardiographic abnormalities in treated infected mice (**Supplementary Fig. S1**). Across groups, recordings showed broadly regular rhythms and preserved repetitive QRS morphology, without evidence of sustained arrhythmias, recurrent ectopic activity, gross QRS widening, obvious atrioventricular or intraventricular conduction defects, or reproducible ST-T/repolarization abnormalities. Overall, these findings are in line with the echocardiographic results and indicate that overt chronic cardiac electrical abnormalities were not evident under the present qualitative assessment at 105 dpi.

### Histopathological evaluation of H&E-stained liver sections revealed a more preserved hepatic morphology in the BZ-MDDS-treated groups than in mice receiving free BZ.

**As expected**, NI mice exhibited conserved parenchymal hepatocyte architecture, no inflammatory infiltrate, no vessel congestion, and diffuse cytoplasmic vacuolization compatible with mild steatotic or hydropic changes. A similar overall pattern was observed in the BZ-MDDS + SS group, which showed a low inflammatory score (-/+), preserved architecture, mild steatosis, and alterations in 3 of 6 mice, mainly subtle cytoplasmic hydropic changes and one case of mild vessel congestion. The BZ-MDDS+APCP group also maintained overall parenchymal organization, with a low inflammatory score (-/+), mild steatosis, and alterations in 4 of 6 mice; subtle hydropic changes and isolated mild vessel congestion were also observed. In contrast, mice treated with free BZ showed more evident hepatic alterations. The BZ + SS group displayed preserved overall architecture but mild inflammatory infiltrate, mild steatosis, mild vessel congestion, and the presence of amastigote nests, with alterations present in all animals analyzed. The BZ+APCP group showed the highest inflammatory score (++), mild architectural alterations, focal necrotic changes, mild steatosis, and amastigote nests, and alterations were also present in all animals analyzed. Mild steatosis was observed in all groups. Overall, these findings indicate that liver morphology remained better preserved in the BZ-MDDS-treated groups, whereas the free BZ regimens—particularly BZ+APCP—were associated with more evident inflammatory and parasitism-related alterations, a pattern that is directionally consistent with the hepatic biochemical signal observed in the present study and with previous reports suggesting that both *T. cruzi* infection and BZ exposure may contribute to hepatic injury and remodeling [12, 23].

Histological findings are presented descriptively. Fibrosis was not observed in any animal. Inflammatory infiltrate, architectural alterations, necrosis, steatosis, and vessel congestion were scored as absent (-), focal (1+), mild (2+), extensive (3+), or diffuse/severe (4+). The complete results are presented in **Supplementary Table S1**.

## 5. DISCUSSION

Overall, the present findings show that the tested reduced-dose intermittent regimen was sufficient to preserve survival in this model of experimental *T. cruzi* infection, even when the cumulative drug burden is substantially reduced. The fact that all BZ-based regimens preserved a marked survival benefit at 50 mg/kg every 2 days for 20 doses is important not only as an efficacy signal per se, but also because it is consistent with the growing evidence showing that lower BZ exposure can still maintain robust anti-*T. cruzi* activity in mice, as previously reported by Bustamante et al. [11] and Perin et al. [14]. Within this framework, the comparable behavior of free BZ and BZ-MDDS becomes pharmacologically relevant, because it suggests that drug incorporation into the carrier system preserved therapeutic performance under a reduced-intensity regimen rather than diluting it, which is fully aligned with our previous observations [12] and with the formulation rationale developed by Garcia et al. [17]. At the same time, the absence of clear differences in terminal blood parasitemia and cardiac T. cruzi DNA among treated groups suggests that, under the present conditions and with the endpoints assessed here, reduced-dose chemotherapy was sufficient to prevent the lethal outcome without revealing major late parasitological differences between formulations. This point deserves some caution, however, because in CD blood parasitemia and tissue persistence are not necessarily superimposable readouts, and residual infection may remain detectable in tissues even when conventional parasitological assessments are markedly improved, as discussed by Tarleton [29], demonstrated experimentally by Martins, et al. [30], and also suggested by our previous APCP study, in which blood parasitemia was unchanged whereas cardiac and hepatic parasite burden were reduced [16]. From this perspective, the lack of a clearly resolved APCP-associated signal at 120 dpi should probably be interpreted less as evidence against biological activity and more as a reflection of the current experimental setting, in which APCP was introduced later and always on top of an already highly effective BZ-based regimen. An additional and structural explanation is that the benznidazole regimen itself already achieved near-maximal efficacy, with survival and parasitological control approaching NI levels; this ceiling effect would limit the residual variance available to resolve an adjunctive APCP effect, independent of its administration timing

A further implication of the present study is pharmacological rather than purely parasitological. The preservation of a marked survival benefit under a regimen of 50 mg/kg every 2 days for 20 doses suggests that the BZ exposure required to maintain therapeutic activity may be lower than that implied by more intensive schedules. This interpretation is consistent with previous experimental evidence showing that reduced or intermittent BZ regimens can retain substantial anti-*T. cruzi* efficacy [11, 14], and it is also relevant in light of the long-recognized limitations of BZ therapy, particularly treatment duration, cumulative toxicity, and tolerability in chronic infection [31]. Within this framework, the comparable behavior of free BZ and BZ-MDDS becomes especially meaningful, because it indicates that incorporation of the drug into the carrier system did not compromise therapeutic performance under conditions of reduced drug burden. On the contrary, it supports the pharmacological rationale that controlled delivery may preserve efficacy while potentially improving the safety profile [12, 17]. Thus, the present data do not simply indicate that a lower BZ regimen remained active but suggest that therapeutic benefit can be maintained even when drug exposure is reduced and administered intermittently, and that this benefit is not lost when BZ is delivered through the MDDS platform.

A point that deserves consideration is the challenge of defining treatment efficacy and, particularly, parasitological cure in experimental *T. cruzi* infection. In murine models, cure is rarely supported by a single readout and is instead inferred from a combination of approaches, including cyclophosphamide-induced reactivation, parasite detection in blood or tissues, and, in some settings, immunological surrogate markers. In this regard, studies by Bustamante and colleagues established that BZ-induced cure in C57BL/6 mice is accompanied not only by the absence of detectable parasites after immunosuppression, but also by a contraction of parasite-specific CD8 + T cells and a shift toward a central memory phenotype detected with MHC-peptide tetramers [11, 18]. However, the applicability of this type of immune biomarker is not necessarily uniform across experimental settings, since the magnitude and hierarchy of parasite-specific CD8 + T-cell responses are strongly shaped by host genetic background and epitope immunodominance, as shown in BALB/c- and C57BL/6-based systems by Tzelepis, et al. [32] and Rosenberg, et al. [33]. In the present study, we attempted to incorporate this tetramer-based approach as a complementary readout, but we were unable to detect a measurable signal in blood at 105 dpi (**Supplementary Fig. S2**). This should be considered a methodological limitation, but it also highlights that immune biomarkers of cure in experimental CD may require model-specific optimization rather than simple extrapolation from previously validated C57BL/6 systems.

A second relevant aspect of the present study is that, under this reduced-dose intermittent regimen, the residual signal was more evident at the hepatic than at the cardiac level. Indeed, GPT was the most sensitive serum marker in the present setting, and this biochemical pattern was reinforced by the liver histology. Taken together, these findings suggest that, at least under the present experimental conditions, the liver remained a more informative compartment for detecting residual treatment-associated differences. This point is relevant because it indicates that, even when survival and terminal blood and heart parasitological readouts are broadly comparable, formulation may still influence tissue-level outcome. The histological findings strengthen this interpretation. Both BZ-MDDS groups largely preserved parenchymal organization and showed only mild cytoplasmic hydropic change, together with the lowest inflammatory scores. Furthermore, the absence of fibrosis and the presence of only mild steatosis across groups indicate that the hepatic phenotype was not one of advanced remodeling, but rather one of relatively subtle yet consistent differences in inflammatory and parenchymal preservation. In other words, the main distinction among regimens was not the development of severe structural liver injury, but the degree to which hepatic architecture remained preserved. This interpretation is in line with our previous report showing that BZ-loaded MDDS improved safety while maintaining anti-*T. cruzi* efficacy, particularly at the hepatic level [12]. It is also consistent with the broader literature showing that both *T. cruzi* infection and BZ exposure can contribute to hepatic injury and remodeling. In particular, Novaes et al. showed that infection and BZ therapy independently stimulated oxidative stress and structural pathological remodeling in mouse liver [23], whereas Davies et al. highlighted the usefulness of hepatic biomarkers and histology as safety readouts in experimental anti-trypanosomal therapy and confirmed that BZ itself is not biologically inert at the hepatic level [20]. In addition, in a murine sepsis model, BZ modified hepatic inflammatory and redox pathways supporting the idea that liver outcome likely reflects not only parasite persistence but also drug–host interactions [34]. Taken together, these findings suggest that, under the present low-dose intermittent schedule, BZ-MDDS was associated with a generally more preserved liver profile by both biochemical and histological criteria, although this pattern was not entirely uniform because GPT elevation was also observed in the BZ-MDDS+APCP group. From a pharmacological perspective, this pattern is relevant because improving tissue-level safety while maintaining therapeutic activity is one of the main rationales for using a drug-delivery system. At the same time, because infected untreated mice were not available at the terminal time point and liver PCR was not performed, the present data should still be interpreted as evidence of differential hepatic involvement rather than as definitive proof of reduced residual hepatic parasitism.

A further issue raised by the present study is that cardiac findings remained relatively subtle according to conventional echocardiographic criteria, whereas selected Doppler-derived flow parameters were still altered in some treatment groups. In the context of CD cardiac involvement may evolve heterogeneously and echocardiographic abnormalities do not always emerge as uniform deterioration of conventional systolic indexes, particularly in earlier or less advanced stages of myocardial damage [27, 28, 35]. Previous studies have shown that Doppler- and tissue Doppler-based methods may detect ventricular abnormalities in CD even when more conventional measurements remain preserved, including pseudonormal diastolic dysfunction, early right ventricular dysfunction, and reduced myocardial deformation in asymptomatic or minimally affected patients [36–38]. Within this framework, the lower pulmonary and aortic VTI values observed here may be better interpreted as residual hemodynamic differences than as evidence of frank chronic ventricular dysfunction. The qualitative ECG findings point in the same direction. No treatment group showed a consistent pattern of Chagas-compatible electrocardiographic abnormalities, and sustained arrhythmias, recurrent ectopic activity, gross QRS widening, obvious conduction defects, or reproducible ST-T abnormalities were not identified. This is relevant because electrical and conduction abnormalities are among the main manifestations of CCD and are closely linked to disease progression in the clinical setting [27, 28, 39]. At the same time, this result should be interpreted with caution, since the ECG assessment was qualitative rather than quantitative and therefore may not have captured more subtle interval abnormalities. In this regard, it should be noted that quantitative ECG analysis was not performed because the recordings were intended for qualitative screening of major Chagas-compatible abnormalities rather than for standardized interval-based measurements. Furthermore, because infected untreated mice were not available at the time of cardiac assessment, the comparison was necessarily restricted to NI and surviving treated groups. The current cardiac findings also need to be interpreted in light of our previous studies. Our earlier BZ-MDDS study did not include cardiac functional assessment, whereas the APCP study showed improvement in chronic ECG parameters under APCP monotherapy [12, 16]. By contrast, in the present work, APCP was administered later, and always on top of an already effective BZ-based regimen. Under those conditions, any additional cardiac effect of CD73 inhibition may have been harder to resolve. Taken together, the echocardiographic and qualitative ECG data suggest that late cardiac alterations were modest and heterogeneous in the surviving treated animals, with no evidence of overt chronic electrical or mechanical dysfunction.

Finally, among the organ-weight readouts evaluated, visceral adipose tissue was the only parameter that remained clearly sensitive to treatment-associated differences at the terminal time point. This finding should be interpreted cautiously since adipose parasitism was not directly measured in the present study. Even so, it is biologically plausible in the context of *T. cruzi* infection. Experimental studies have shown that adipocytes are direct targets of *T. cruzi* and that adipose tissue can serve as a site of parasite persistence [25, 26]. In addition, early infection has been associated with high parasite load in adipose tissue, increased macrophage infiltration, reduced lipid accumulation, smaller adipocyte size, and lower fat mass [40]. Thus, the reduction in visceral adipose tissue observed in some treated groups may indicate that adipose tissue remains a biologically responsive compartment even when the overall terminal parasitological profile appears broadly similar across regimens, although, in the absence of direct assessment of adipose parasitism, the reduction in visceral adipose tissue observed in the treated groups should be considered an exploratory finding.

## 6. CONCLUSIONS

In conclusion, the present study shows that a reduced intermittent BZ regimen (50 mg/kg every 2 days for 20 doses) preserved a marked survival benefit in experimental *T. cruzi* infection. Under these conditions, both free BZ and BZ-loaded MDDS showed broadly comparable terminal blood parasitemia and cardiac parasite DNA profiles, indicating that drug incorporation into the carrier system did not compromise therapeutic activity. It should be noted that this conclusion refers specifically to comparability between formulations within the reduced-dose regimen tested here, since a concurrent standard-dose benznidazole arm was not included in the present design; the broader claim that efficacy is preserved relative to conventional dosing rests on comparison with previously published data rather than on a direct internal control. Although adjunctive APCP did not produce a clearly distinguishable terminal parasitological advantage, this result should be interpreted in the context of its later administration and its use on top of an already effective BZ-based regimen. A relevant difference among treatments emerged at the hepatic level, where BZ-MDDS was associated with a more preserved biochemical and histological liver profile than free BZ. Taken together, these findings support the view that therapeutic benefit can be maintained under a lower-intensity BZ schedule and that MDDS delivery may offer a favorable balance between preserved efficacy and improved tissue-level safety.

## 7. LIMITATIONS

This study has several limitations. First, infected untreated mice were not available at the terminal time point because none survived to 120 dpi; therefore, late comparisons were necessarily restricted to non-infected controls and surviving treated groups. Second, treatment response was assessed primarily using survival and terminal parasitological endpoints, and although a tetramer-based immune biomarker approach was explored, no measurable signal could be detected in blood at 105 dpi. Third, liver injury was evaluated by biochemical markers and histology, but molecular assessment of hepatic parasitism was not performed, which limits interpretation of the relationship between amastigote nests and residual parasite burden. Fourth, surface ECG recordings were assessed qualitatively because P-wave onset and the terminal boundaries of the QRS and T waves were not reproducibly identifiable across all animals. Given the recognized limitations of interval measurement in murine surface ECGs, PR/PQ, QRS, and QT/QTc were not quantified; therefore, subtle between-group differences in these parameters cannot be excluded [41, 42]. Finally, the APCP regimen used here differed from previous studies in timing and therapeutic context, since it was administered later and always in combination with BZ-based treatment; accordingly, the present design may have underestimated a more discrete adjunctive effect of CD73 inhibition. In addition, and independently of this timing consideration, the BZ regimen itself already produced near-maximal survival and parasitological control, which constitutes a ceiling effect that would limit the statistical power to detect any additional benefit of APCP regardless of when or how it was administered. Finally, in order to reduce the number of animals employed, the present design did not include a concurrent standard-dose BZ arm; therefore, claims that efficacy is preserved relative to conventional dosing rest on comparison with our previously published data rather than on a direct internal control.

## Supplementary Material

Supplementary Files

This is a list of supplementary files associated with this preprint. Click to download.

• SupplementaryMaterial.pdf

## Figures and Tables

**Figure 6:**
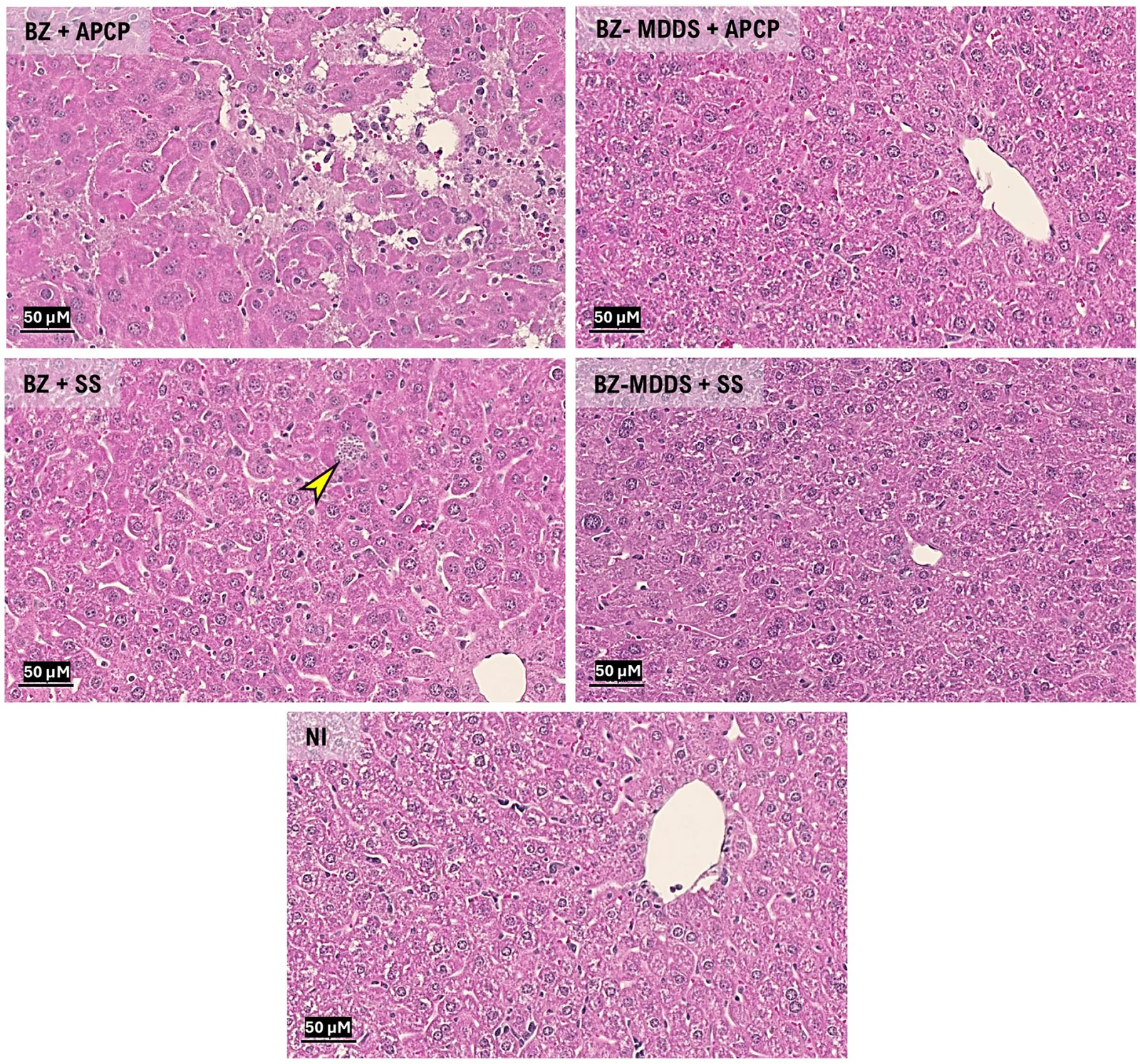
Liver histology and inflammatory index: Representative photomicrographs of H&E-stained liver tissue sections of different experimental groups. The analysis was performed at 120 dpi. The arrow indicates an amastigote nest.

**Table 1:** Semi-quantitative determination of inflammatory foci as well as histological analysis of hepatic tissue damage. The analysis was performed at 120 dpi.

Group	n	Inflammatory infiltrate (n/N, severity)	Architecture alterations (n/N)	Necrosis (n/N)	Steatosis (n/N)	Vessel congestion (n/N)	Amastigote nests (n/N)
NI	5	0/5	0/5	0/5	4/5 (2+)	0/5	0/5
BZ-MDDS + SS	6	3/6 (all 1+)	0/6	0/6	4/6 (2+)	1/6 (2+)	0/6
BZ-MDDS+APCP	6	4/6 (all 1+)	0/6	0/6	4/6 (2+)	1/6 (1+)	0/6
BZ + SS	5	5/5 (3 with 1+, 2 with 2+)	0/5	0/5	2/5 (2+)	3/5 (2 with 1+, 1 with 2+)	3/5
BZ+APCP	5	5/5 (3 with 1+, 2 with 2+)	2/5 (2+)	2/5 (1 with 1+, 1 with 2+)	2/5 (2+)	0/5	2/5

## Data Availability

The datasets generated during and/or analyzed during the current study are available from the corresponding author upon reasonable request
